# oxBS-450K: A method for analysing hydroxymethylation using 450K BeadChips

**DOI:** 10.1016/j.ymeth.2014.08.009

**Published:** 2015-01-15

**Authors:** Sabrina K. Stewart, Tiffany J. Morris, Paul Guilhamon, Harry Bulstrode, Martin Bachman, Shankar Balasubramanian, Stephan Beck

**Affiliations:** a Department of Cancer Biology, UCL Cancer Institute, University College London, London WC1E 6BT, UK; b Scottish Centre for Regenerative Medicine, Edinburgh EH16 4UU, UK; c Department of Chemistry, University of Cambridge, Cambridge CB2 1EW, UK

**Keywords:** Hydroxymethylation, DNA methylation, 450K BeadChip, Oxidation, Bisulfite conversion, Epigenetics

## Abstract

DNA methylation analysis has become an integral part of biomedical research. For high-throughput applications such as epigenome-wide association studies, the Infinium HumanMethylation450 (450K) BeadChip is currently the platform of choice. However, BeadChip processing relies on traditional bisulfite (BS) based protocols which cannot discriminate between 5-methylcytosine (5mC) and 5-hydroxymethylcytosine (5hmC). Here, we report the adaptation of the recently developed oxidative bisulfite (oxBS) chemistry to specifically detect both 5mC and 5hmC in a single workflow using 450K BeadChips, termed oxBS-450K. Supported by validation using mass spectrometry and pyrosequencing, we demonstrate reproducible (*R*^2^ > 0.99) detection of 5hmC in human brain tissue using the optimised oxBS-450K protocol described here.

## Highlights

• A method is presented for 5hmC detection and analysis using Infinium 450K BeadChips.
• The oxBS-450K method can discriminate between 5mC and 5hmC in human gDNA
• 5hmC levels were quantified genome-wide in 3 distinct biological samples.
• The reported 5hmC signal was validated using mass spectrometry and pyrosequencing.
• The effects of differing amounts of input DNA on final 5hmC call rate are discussed.

## 1 Introduction

DNA methylation studies have been traditionally concerned with 5-methylcytosine (5mC), mostly in the context of CpG dinucleotides, which until recently was the only epigenetic modification of DNA with known biological function. The rediscovery of 5-hydroxymethylcytosine (5hmC) in mammalian DNA has opened up an exciting new area of epigenetic research concerning the potential role of 5hmC in both normal development and disease progression [1], [2]. 5hmC has been proposed to be an intermediate in an active DNA demethylation pathway and may also be a stable epigenetic modification in its own right, contributing unique regulatory functions to the epigenome [3].

Current methods for profiling 5hmC at a genome-wide level rely largely on sequencing-based protocols that are both costly and time-consuming. In addition, they require a high degree of expertise with the chosen assay for successful implementation. The Infinium HumanMethylation450 BeadChip is a widely-used, robust and reliable tool for DNA methylation profiling [4], [5], suitable for high-throughput sample processing and is therefore the platform of choice for current epigenome-wide association studies [6], [7]. The 450K array interrogates over 480,000 CpG sites in the human genome, making it an appropriate tool for 5hmC detection as this modification has been reported to occur exclusively in the CpG context in both mammalian embryonic stem cells and frontal cortex tissue [8], [9], [10]. However, current workflows for this microarray rely on sodium bisulfite conversion of DNA, which discriminates between methylated and unmethylated cytosine bases within the genome but cannot differentiate between 5mC and 5hmC [11].

The recent development of oxidative bisulfite (oxBS) chemistry presents an opportunity to adapt bisulfite-based 5mC profiling technology for 5hmC detection [9]. Here we describe low- and high-input protocols for genome-wide profiling of 5hmC by coupling oxBS chemistry and the Infinium 450K BeadChip (oxBS-450K). Alternative approaches also using oxBS or Tet-assisted bisulfite (TAB) chemistries have been developed by Field et al. [12] and Chopra et al. [13], respectively. In brief, oxBS involves a selective oxidation step prior to bisulfite conversion of genomic DNA that converts 5hmC residues to 5-formylcytosine (5fC) with high efficiency. 5fC behaves in a manner analogous to unmodified cytosine under bisulfite conditions, allowing only true 5mC positions to be detected as cytosine after oxidative bisulfite conversion and PCR amplification of the DNA sample. Subtraction of oxBS-generated methylation profiles from standard BS-only methylation profiles allows for the detection of hydroxymethylated cytosine positions within the genome. Whilst traditional bisulfite conversion leads to potential overestimation of 5mC levels in a given sample due to the presence of 5hmC, the oxBS-450K method generates a more accurate 5mC profile by removing the confounding factor of 5hmC.

Global 5hmC levels are known to vary considerably between tissue types and have been found to be highest in the brain [14], [15] and so the distribution of 5hmC in this tissue may be of particular interest. Here, we show that the Infinium 450K can be used to successfully detect 5hmC in three distinct genomic DNA samples (two brain and one whole blood) by comparison of BS- and oxBS-450K datasets and provide validation of our reported 5hmC signal using independent technologies.

## 2 Materials and methods

### 2.1 DNA samples

All procedures followed are in accordance with the ethical standards of the Helsinki declaration (1964, 2008) of the World Medical Association. Human brain genomic DNA from a 33 year-old male was obtained from AMSBIO (catalogue #D1234035, lot #B701205; referred to as ‘brain1’). Human frontal cortex DNA was collected according to Ethical Approval obtained from the Berkshire Local Research Ethics Committee (REC 08/H0505/165), with informed written consent (referred to as ‘brain2’). Pooled (male and female) whole blood DNA was obtained from blood donations of volunteers from the UCL Cancer Institute.

### 2.2 Oxidative bisulfite (oxBS) and bisulfite-only (BS) conversion workflows

We tested two protocols (low and high input) and report the results for both to highlight critical steps and provide insights into their dynamics.

#### 2.2.1 Low-input oxBS-450K protocol

1 μg DNA per sample was processed using a trial version of the TrueMethyl® 24 kit (CEGX). Samples were split evenly into two aliquots of ∼500 ng processed through either the BS-only or oxBS conversion workflow. Each aliquot was subjected to an initial buffer exchange step using a spin column format and eluted in ultra-pure water. The full eluate (∼22 μl per sample) was carried forward. Samples were denatured using the provided denaturing solution for 30 min at 37 °C, in a total reaction volume of 24 μl and immediately taken forward to oxidation. 1 μl of the provided oxidant solution was added to each sample undergoing the oxBS workflow only (1 μl of ultra-pure water was added to BS-only samples for mock oxidation). All samples were incubated for 30 min at 40 °C. Oxidised samples were equilibrated to room temperature before proceeding immediately to bisulfite conversion. Bisulfite reagent solution was prepared as described in the TrueMethyl® protocol and 170 μl was added to each 25 μl oxidation reaction mixture. Finally, 5 μl of bisulfite additive was added to each reaction, bringing the total volume to 200 μl. All reactions were incubated using bisulfite-specific thermal cycling conditions (see Supplementary information). Converted DNAs were purified using the provided spin columns and 4 μl of each sample eluate was used as input into the Infinium 450K array.

#### 2.2.2 High-input oxBS-450K protocol

4 μg DNA per sample was processed using the TrueMethyl® protocol for 450K analysis (Version 1.1, CEGX™). The protocol was performed as described, with 2 minor alterations. First, the initial shearing step of DNA was performed using a Bioruptor (Diagenode™). The DNA was diluted into a total volume of 50 μl in 1.5 ml microtubes and sonicated for 30 s on setting ‘H’. 10 μl was set aside for visualisation on a 2% agarose gel to confirm correct DNA fragmentation (all ⩽10 kb). Secondly, the remaining 40 μl (∼3.2 ug) of sheared DNA was split into 2 × 20 μl aliquots and purified using Agencourt AMPure XP® beads using a modified protocol (http://www.cambridge-epigenetix.com/uploads/files/TrueMethyl24_UGuide.pdf, TrueMethyl® 24 kit user guide, CEGX™). Each sample was eluted in 20 μl ultra-pure water and then processed either through the oxBS or BS-only workflow as outlined in the TrueMethyl® user guide. 7 μl of the final eluate was used as input into the Infinium 450K array, made possible by adding 1 μl of 0.4 N NaOH to each sample rather than the standard 4 μl of 0.1 N NaOH when setting up the MSA4 plate.

### 2.3 450K BeadChip processing and data analysis

450K BeadChips were processed by UCL Genomics following the manufacturer’s recommendations and data was analysed using the Chip Analysis Methylation Pipeline (ChAMP) implemented in *R* and available from Bioconductor [16]. Briefly, raw IDAT files were used as input data and probes were filtered by their raw intensity values using a detection *p*-value threshold of 0.01. Probes corresponding to the X and Y chromosomes were removed from the dataset as both male and female samples were being analysed. The data was then normalised using the SWAN method [17], producing a final dataset containing 432,056 probes and their corresponding beta values for each sample for further downstream analysis. The *R* package *pvclust* was used for multiscale bootstrap resampling (1000 replications) for hierarchical cluster analysis.

### 2.4 Oxidative bisulfite pyrosequencing (oxBS-pyroseq)

50 bp windows containing at least 2 probes showing a hydroxymethylation level of 30% or higher (based on the AMSBIO brain dataset) were selected for validation by oxBS-pyroseq. Primers were designed using PyroMark Assay Design 2.0 software (see supplementary information for sequences and probe IDs). Two aliquots of AMSBIO human brain DNA underwent either oxidative bisulfite or bisulfite-only conversion (as detailed in Section 2.2) and were PCR amplified using a Pyromark PCR kit (Qiagen, cat. #978703) under the following thermocycling conditions: 95 °C for 15 min, (94 °C for 30 s, 66 °C (−0.5 °C per cycle) for 30 s, 72 °C for 30 s) × 13 cycles, (94 °C for 30 s, 56 °C for 30 s, 72 °C for 30 s) × 50 cycles, 72 °C for 10 min. Assays were run on a PyroMark Q96 ID system and results were analysed using Pyro Q-CpG 1.0.9 software. Methylation scores at each CpG site were normalised against a linear calibration curve and 5hmC levels calculated by subtraction of oxBS from BS-only methylation calls.

### 2.5 Mass spectrometry

500 ng of genomic DNA was incubated with 5 U of DNA Degradase Plus (Zymo Research) at 37 °C for 3 h. The resulting mixture of 2′-deoxynucleosides was analysed on a Triple Quad 6500 mass spectrometer (AB Sciex) fitted with an Infinity 1290 LC system (Agilent) and an Acquity UPLC HSS T3 column (Waters), using a gradient of water and acetonitrile with 0.1% formic acid. External calibration was performed using synthetic standards, and for accurate quantification, all samples and standards were spiked with isotopically labelled nucleosides. 5mC and 5hmC levels are expressed as a percentage of total cytosines.

## 3 Results and discussion

Global 5hmC levels are known to be lower than 5mC levels and more varied between different tissues [14], [15]. To test the oxBS-450K protocol, we therefore selected tissues expected to have high (two types of brain tissue) and low (whole blood) levels of 5hmC and confirmed their percentage by quantitative LC–MS to be 0.039% for whole blood and 0.924% and 1.107% for the two brain samples, respectively. In comparison, the corresponding 5mC levels were ∼4% in all three samples.

### 3.1 Identification of 5hmC

5hmC detection was achieved by identifying differentially methylated CpG sites between the BS- and oxBS-treated replicates within each sample set. The beta value associated with each probe on an array reflects the methylation level at that particular location on a scale of 0–1, where 0 is unmethylated and 1 is fully methylated.

Beta values resulting from BS treatment represent the total methylation score (5mC and 5hmC) as both cytosine modifications undergo bisulfite conversion to uracil with comparable efficiency. In contrast, beta values resulting from oxBS treatment represent only the 5mC level at the corresponding probe locations. Therefore normalised beta values were used to calculate delta beta (Δ*β*) values for each probe by subtraction of the oxBS beta value from the BS-only beta value. The Δ*β* score is a reflection of the 5hmC level at each particular probe location.

**Fig. 1:**
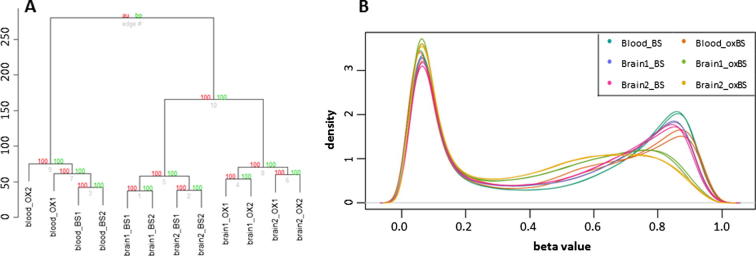
QC plots for 450K dataset generated using low-input protocol. (A) Unsupervised hierarchical clustering of all 12 samples show clear separation between tissues types (brain and blood) and also between BS-only and oxBS conversion. “Au” figures, in red, refer to approximately unbiased *p*-values (%); “bp” numbers, in green, refer to bootstrap *p*-values (%). (B) Density plot of normalised beta values for all 12 samples. Beta values show a bimodal distribution for BS-only conversion samples, whilst a left-skewed methylated peak is seen in the oxBS conversion samples, corresponding to lower global methylation levels.

Unsupervised hierarchical clustering of this dataset showed a clear separation between blood, brain1 and brain2 and also between treatment options, with both BS-only and oxBS-treated replicates of each sample clustering in pairs (Fig. 1A). The corresponding density profiles show a clear reduction of methylation signals after oxBS treatment (Fig. 1B).

**Fig. 2:**
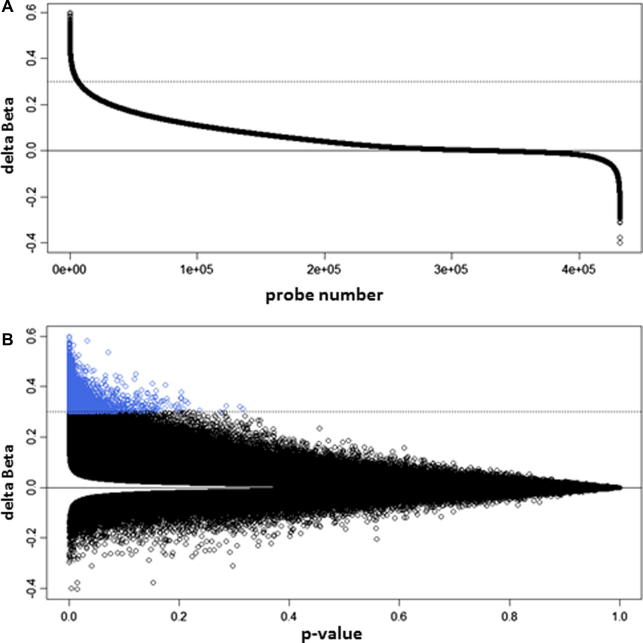
Delta beta (Δ*β*) distribution in brain1 sample. (A) Δ*β* distribution across all probes after filtering. (B) Δ*β* distribution against their associated *p*-value. Data points highlighted in blue indicate those probes called as hydroxymethylated (Δ*β* ⩾ 0.3 and corresponding *p*-value <0.05).

**Fig. 3:**
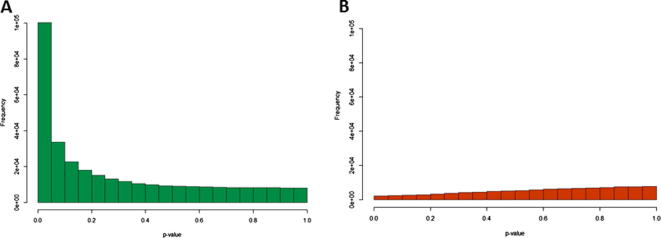
Frequency distribution of all probes according to their associated *p*-value. (A) Frequency distribution of all probes associated with a Δ*β* > 0, or potential sites of 5hmC. (B) Frequency distribution of all probes associated with a Δ*β* < 0, i.e. false positives.

Using this method, both positive and negative Δ*β* scores were calculated. Positive Δ*β* values represent potential sites of 5hmC; negative Δ*β* values, or ‘hypermethylation’ in the oxBS-treated sample, were not expected as the oxidation reaction is unidirectional and thus are likely to reflect background noise generated by this method. Fig. 2A shows the Δ*β* distribution across all probes for the brain1 sample, displaying significantly more positive Δ*β* scores than negative, as expected. Less than 3% of all negative Δ*β* scores correspond to a 5hmC level of more than 10%, however over 30% of all positive Δ*β* scores correspond to a 5hmC level over 10%. In addition, Fig. 2B shows that the largest Δ*β* scores are associated with the most significant *p*-values and that significance falls away as the Δ*β* values approach zero, as expected. Furthermore, frequency plots of all probes according to their associated *p*-values (Fig. 3) show that the majority of Δ*β* values above zero represent significant differences in methylation score (*p* ⩽ 0.05). In contrast, the *p*-values associated with negative Δ*β* scores are distributed across the entire range (0–1), with increasing numbers of probes associated with higher *p*-values. This is consistent with the idea that the negative Δ*β* scores represent false differences in methylation score between the paired BS-only and oxBS datasets.

There are various ways to define an appropriate Δ*β* threshold to identify a probe-set of significantly hydroxymethylated cytosines, including shrinkage estimation of variances, but this approach requires each sample to be run in multiple (3–4) replicates resulting in extra costs [12]. As the amount of available genomic DNA is often limiting, we based our strategy on running two replicates per sample and opted for using a conservative threshold of Δ*β* ⩾ 0.3, or minimum 30% 5hmC, followed by validation. Using this approach, we identified 6578 and 7692 probes in our brain1 and brain2 samples, respectively, in contrast to just 801 probes in whole blood DNA. Inclusion of a third technical replicate of the brain1 sample, however, increased the statistical power of the dataset by allowing the Δ*β* calculations to reach statistical significance. We were able to use a BH-adjusted *p*-value threshold of 0.05 to call 5hmC, which dramatically increased the probe number because any significant Δ*β* values in the range of 0–30% 5hmC could now be identified alongside the more highly hydroxymethylated probes, resulting in the detection of 64,720 significant probes in total. Of these, only 117 sites were associated with negative (but nevertheless significant) Δ*β* scores, corresponding to a false discovery rate of less than 0.002. 5hmC levels fell within the range of 4–59%, with a mean level of 18% 5hmC across all positive 5hmC probe sites whilst all negative Δ*β* scores corresponded to less than 20%.

**Fig. 4:**
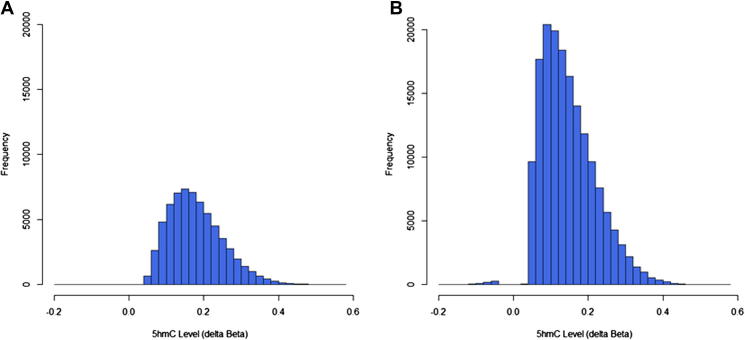
5hmC levels across all significantly hydroxymethylated probes. (A) Frequency distribution of 5hmC levels, expressed as a Δ*β* score, across all 64,720 5hmC sites in the brain1 sample processed in triplicate with the initial protocol. (B) Frequency distribution of 5hmC levels, expressed as a Δ*β* score, across all 165,495 5hmC sites in the brain1 sample, processed in duplicate with the optimised protocol. Both protocols detect similar ranges.

While the low-input oxBS-450K protocol only requires 1 μg starting DNA, the high-input protocol requires 4 μg DNA but results in much improved sensitivity and reproducibility. Using the same detection *p*-value threshold of 0.01 as for the low-input protocol to filter probes, we found that the technical replicates showed an improved correlation of *R*^2^ > 0.99 compared to *R*^2^ > 0.95 for the low-input protocol, comparable to current ‘gold-standard’ bisulfite conversion protocols. Comparison of BS-only and oxBS datasets using just two replicates per sample was sufficient to call 165,495 5hmC sites (BH-adjusted *p*-value < 0.05) with just 627 false positive probes associated with a negative Δ*β* score (Fig. 4). 5hmC levels were detected in the range of 4–57%, with a mean 5hmC level of 15%. In contrast, negative Δ*β* values all fell below 20%.

Analysis of the significant but negative Δ*β* probes between the low- and high-input protocols for the brain1 sample suggests that the locations of these negative sites are random: 117 sites were called by the low-input (3-replicate) method compared to 627 with the high-input method, yet only 8 negative Δ*β* sites were found in common between the two (the increase in negative probe number is a reflection of an overall increase in the number of significant probes identified using the high-input method). Despite showing statistically significant *β* value differences, these pseudo false positives cannot reflect true differences in methylation level at these probe sites and should be filtered out before further downstream data analysis.

**Supplementary Fig. 1:**
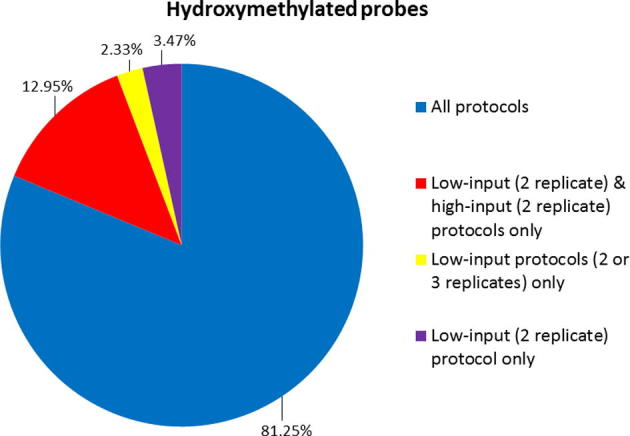
5hmC probe overlap between the three analysis methods for brain1. Overlap is shown for the 6578 probes called as hydroxymethylated using the low-input (2 replicate) protocol. Of these, 81.3% remained significantly hydroxymethylated both when the replicate number was increased and when the experiment was repeated using the high-input protocol. Only 3.5% of these probes were unable to reach significance when either replicate number was increased or when higher amounts of input DNA were used.

Comparison of the 5hmC-containing probes identified by the different protocols shows that overlap is high, supporting our conclusion that these are sites of genuine 5hmC. 83.6% of the 5hmC-containing sites initially called by the low-input, 2-replicate method remained significantly hydroxymethylated with the addition of a third replicate (5498 of 6578). This figure increases to 94.2% when comparison is made to the high-input, 2-replicate dataset. Moreover, only 3.5% of the 5hmC probes called by the low-input method fail to reach significance when either replicate number or DNA input is increased, suggesting that the false positive rate of our low-input, 2-replicate method is fairly low (Supplementary Fig. 1).

**Fig. 5:**
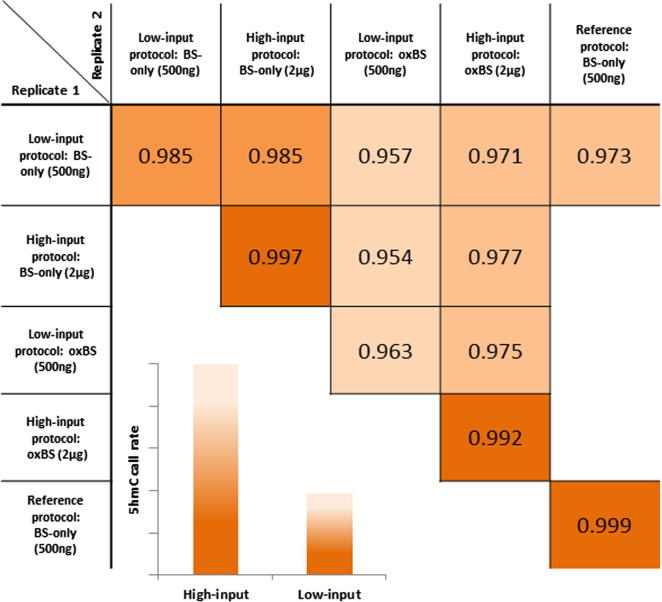
Correlation matrix for low- and high-input oxBS-450K protocols. The high-input protocol results in correlations of *R*^2^ > 0.99 for both oxBS and BS-only replicates, comparable to that of an alternative commercially-available bisulfite conversion kit (referred to as the reference protocol). The amount of input DNA needed per conversion workflow is indicated in the table. Note that processing of at least two replicates per sample through both BS-only and oxBS workflows is required to calculate 5hmC levels. Despite high correlations between replicates for both protocols, the embedded bar chart shows a trend of greatly reduced 5hmC call rate (number of probes identified with significant 5hmC content) when using lower amounts of input DNA.

We conclude that the use of the high-input oxBS-450K protocol on just two replicates results in detection of 5hmC at over twice the number of probes compared to the low-input protocol using three replicates. However, both the mean and range of 5hmC levels detected is very similar in both cases, suggesting that using lower amounts of input DNA does not necessarily affect the quality of the array data produced, but does severely limit the number of probes whose Δ*β* values reach statistical significance. The correlation matrix shown in Fig. 5 is intended to help guide potential users on which protocol to use.

### 3.2 Distribution of 5hmC

**Fig. 6:**
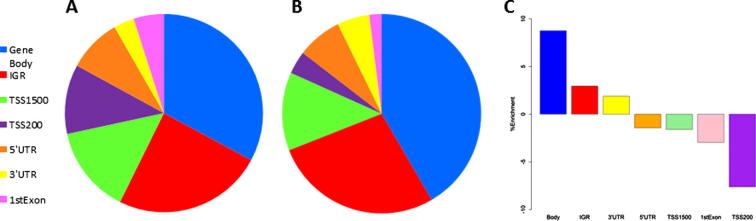
Probe distribution according to genomic features. (A) Distribution of all probes after filtering (432,056 total) on 450K BeadChip. (B) Distribution of hydroxymethylated probes (6578 total) in brain1 sample. (C) Enrichment of 5hmC probes according to various genomic regions. Significant enrichment is seen in the gene body, and to a lesser extent 1500 bp upstream of the TSS. Conversely, significant depletion is seen in the proximal promoter region (TSS200) and 1st exon.

**Supplementary Fig. 2:**
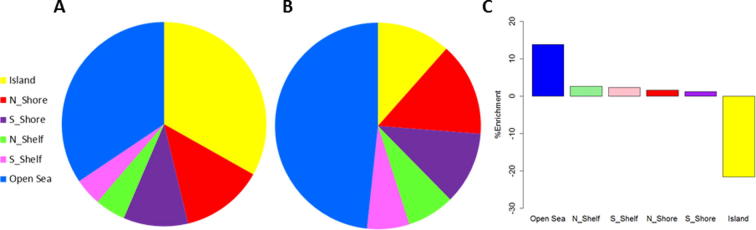
SuProbe distribution according to CpG context. (A) Distribution of all probes after filtering (432,056 total) on 450K BeadChip according to CpG context. (B) Distribution of hydroxymethylated probes (6578 total) in brain1 sample according to CpG context. (C) Relative enrichment of 5hmC probes in differing CpG contexts. Significant enrichment of 5hmC is seen in ‘open sea’ regions of low CpG density, although the most striking observation is the depletion of 5hmC at CpG islands.

We used our probe sets of significant 5hmC sites to investigate the distribution of 5hmC within different genomic features, compared to the overall distribution of probes present on the array. The following categories were used for classification of each probe: 3′UTR region, 5′UTR region, 1st exon, 200 bp window upstream of the TSS (TSS200), 1500 bp window upstream of the TSS (TSS1500), intergenic region or gene body. A permutation test was performed to calculate percentage enrichment for 5hmC within each feature (Fig. 6) and 5hmC was found to be significantly enriched in the gene body in all three DNA samples. This is consistent with previous studies concerning the genomic distribution of 5hmC [13], [18]. Conversely, 5hmC was found to be significantly depleted from promoter regions (defined as within 1500 bp upstream of TSS) and from CpG islands (Supplementary Fig. 2), which agrees with several published studies that describe an inverse correlation between 5hmC and CpG density at the promoter [9], [13], [19].

### 3.3 Validation of 5hmC

**Supplementary Fig. 3:**
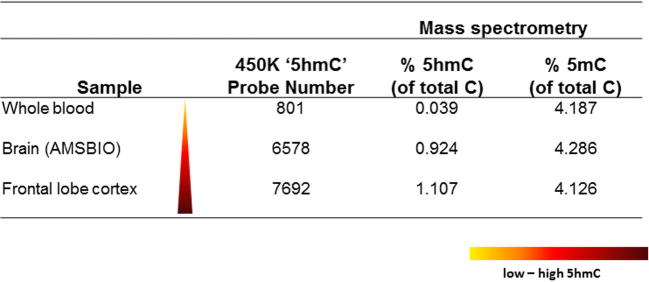
LC–MS quantification of 5mC/5hmC content in 3 DNA samples. The fraction of 5mC appears consistent across all samples, in contrast to the 5hmC content, where nearly 30-fold higher levels are seen in the 2 brain samples as compared to whole blood. This pattern of increased levels of 5hmC in brain is reflected in the 450K dataset (low-input protocol results are shown), where the number of CpG sites containing the highest levels of 5hmC (⩾30%) are around 10-fold higher in brain versus blood.

We used two methods (LC–MS and oxBS-pyroseq) to validate 5hmC identified with oxBS-450K. On the global level, we used quantitative LC–MS to confirm that each of our samples did indeed contain 5hmC levels suitable for detection with oxBS-450K. Using the average of two replicates, we established that the 5hmC levels of both brain samples were similar, at around 1% of total cytosine bases (0.92% in brain1 and 1.11% in brain2), whilst the whole blood sample contained only low levels of 5hmC, at 0.04% of total cytosine as expected. In contrast, 5mC levels in all three samples were broadly consistent at around 4% of total cytosine. The overall pattern of total 5hmC content per sample correlated well with the observed 5hmC signal from the 450K array data, where we observed ten-fold higher numbers of probes with significant 5hmC in the brain as compared to whole blood (Supplementary Fig. 3).

**Supplementary Fig. 4:**
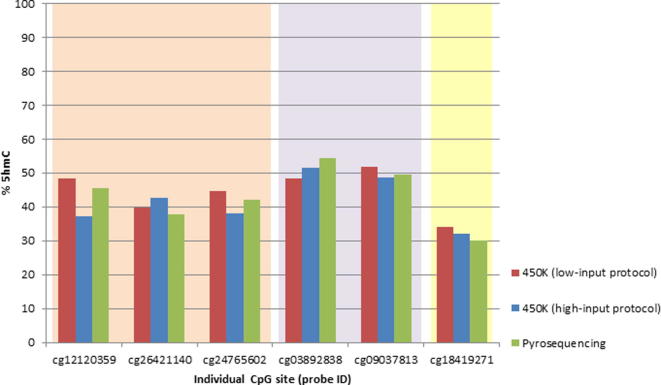
Validation of 5hmC calls using a pyrosequencing assay. 5hmC levels at 6 individual CpG sites distributed across three distinct genomic regions (indicated by orange, purple and yellow shading, respectively) were validated in the brain1 sample. Results are plotted as % 5hmC at each CpG locus as measured by pyrosequencing, low-input 450K and high-input 450K assays.

On the single cytosine level, we used quantitative oxBS-pyroseq to validate selected 5hmC sites identified with oxBS-450K in our brain1 sample. The hydroxymethylation status of six individual CpG sites across three genomic regions was confirmed using this assay (Supplementary Fig. 4), with 5hmC levels differing by less than 10% between the 450K array and pyrosequencing assay. In addition, our validation results suggest that there is little difference in the accuracy of 5hmC calling depending on the 450K protocol used, as both low-input and high-input methods generated 5hmC levels within 10% of the pyrosequencing readout.

## 4 Conclusions

We have shown that oxidative bisulfite chemistry can be combined with the 450K DNA methylation microarray to reliably detect 5hmC and 5mC in the human genome. The oxBS-450K protocol presented here results in highly reproducible technical replicates, comparable to current ‘gold-standard’ bisulfite conversion kits. Our analysis shows that 5hmC can be detected at statistically significant levels at over 30% of all CpG sites interrogated on the array, with a mean 5hmC level of 15% in a human brain sample. Whilst the use of an optimised protocol offers an improvement in the correlation of technical replicates and allows a higher proportion of 5hmC sites to be called with statistical significance, the amount of input DNA required may prove limiting for many users. However, validation using oxBS-pyroseq suggests that accurate 5hmC levels, albeit at fewer sites, can nevertheless be called using a lower input of 1 μg DNA for oxBS-450K analysis.

## Appendix A Supplementary data

Supplementary data 1 Supplementary Table 1. Bisulfite conversion thermal cycler conditions. Supplementary Table 2. Pyrosequencing PCR primers and associated probe IDs.
