# Supplementary material for: oxBS-450K: A method for analysing hydroxymethylation using 450K BeadChips
Source: Methods. 2015 Jan 15;72:9–15. doi: 10.1016/j.ymeth.2014.08.009 (PMC4304834; doi:10.1016/j.ymeth.2014.08.009)
Supplement: Supplementary data 1 — Supplementary Table 1. Bisulfite conversion thermal cycler conditions. Supplementary Table 2. Pyrosequencing PCR primers and associated probe IDs. [file mmc1.docx]

| **Cycling step** | **Temperature (°C)** | **Time (min)** |
| --- | --- | --- |
| Denaturation | 95 | 5 |
| Incubation | 60 | 20 |
| Denaturation | 95 | 5 |
| Incubation | 60 | 40 |
| Denaturation | 95 | 5 |
| Incubation | 60 | 165 |
| Denaturation | 95 | 5 |
| Incubation | 60 | 20 |
| Denaturation | 95 | 5 |
| Incubation | 60 | 40 |
| Denaturation | 95 | 5 |
| Incubation | 60 | 165 |
| HOLD | 4 | indefinitely |

**oxBS-450K: A method for analysing hydroxymethylation using 450K BeadChips**

Supplementary Information

**Supplementary Table 1: Bisulfite conversion thermal cycler conditions.**

|  | **Region 1**  (cg12120359; cg26421140; cg24765602) | **Region 2**  (cg03892838; cg09037813) | **Region 3**  (cg18419271) |
| --- | --- | --- | --- |
| **Forward primer** | GGAGGGTTTATGTTTTTAATATTAATTT | GGGAAGGTGAGTGTTTATAG | TGTTTTTTTGGAGATTTGGGAATTTGT |
| **Reverse primer** | *ACTAAAAAACCCATAATATAATCAATCAAA | *CAATTCCCAATTCCCTTTTCTC | *AAAAACCCTCCCCACTTTTAAT |
| **Sequencing primer** | TGAGAGGAGTTAGAAATATGATTAG | AGGTGAGTGTTTATAGG | ATTTGGGAATTTGTGTTTTTTA |

*Denotes biotinylated primer

**Supplementary Table 2: Pyrosequencing PCR primers and associated probe IDs.**
